# Skin microbiome transplantation and manipulation: Current state of the art

**DOI:** 10.1016/j.csbj.2021.01.001

**Published:** 2021-01-04

**Authors:** Chris Callewaert, Nastassia Knödlseder, Ante Karoglan, Marc Güell, Bernhard Paetzold

**Affiliations:** a Center for Microbial Ecology and Technology, Ghent University, Coupure Links 653, Ghent, Belgium; b Department of Pediatrics, University of California San Diego, 9500 Gilman Drive, La Jolla, CA 92093, USA; c Department of Experimental and Health Sciences, Universitat Pompeu Fabra (UPF), C. Dr. Aiguader 88, 08003 Barcelona, Spain; d Department of Dermatology, University Hospital Magdeburg, University of Magdeburg, Magdeburg, Germany; e S-Biomedic, Turnhoutseweg 30, 2340 Beerse, Belgium

**Keywords:** Skin microbiome, Microbiome transplantation, Microbiome manipulation, Skin microbiome modulation

## Abstract

Many skin conditions are associated with an imbalance in the skin microbiome. In recent years, the skin microbiome has become a hot topic, for both therapeutic and cosmetic purposes. The possibility of manipulating the human skin microbiome to address skin conditions has opened exciting new paths for therapy. Here we review the skin microbiome manipulation strategies, ranging from skin microbiome transplantation, over skin bacteriotherapy to the use of prebiotics, probiotics and postbiotics. We summarize all efforts undertaken to exchange, manipulate, transplant or selectively apply the skin microbiome to date. Multiple microbial groups have been targeted, since they have been proven to be beneficial for skin health. We focus on the most common skin disorders and their associated skin microbiome dysbiosis and we review the existing scientific data and clinical trials undertaken to combat these skin conditions. The skin microbiome represents a novel platform for therapy. Transplantation of a complete microbiome or application of single strains has demonstrated beneficial therapeutic application.

## 1 Introduction

The understanding of humans as hosts for trillions of microorganisms has changed the way we understand and target diseases. We live in a symbiotic relationship with our microbes, and our human cells closely interact and communicate with bacterial, fungal and viral cells. This relationship is typically beneficial, but the symbiotic nature can change if the distribution of certain components of the system changes and can lead to dysbiosis or disease.

Probiotics and thereby indirect microbiome modulation are part of an equally long and successful development. The first treatments based on fermented milk date back as far as 10,000 BCE and developed into modern nutrition supplemental products. Amazonian tribes currently still ferment sugar-rich liquids into non-alcoholic beverages (called chicha or masato) that are converted by spontaneous fermentation using lactobacilli and yeasts [1] and this tradition dates back for ages. While some treatments were used as nutritional supplements for various reasons, others were developed into full drug products. A number of phase III clinical trials were conducted (NCT03244644 and NCT03183128) with positive preliminary results announced.

While the probiotic field is old, many recent developments are driven by technological breakthroughs. The emergence of next-generation sequencing has provided unprecedented insights into microbiome composition. We are no longer bound to the great plate anomaly [2], [3] and decreases in the cost and time of this technique and the emergence of bioinformatics pipelines have enabled fast analysis and a more complete understanding of the present microbiome [4].

A very recent development is genetically engineered probiotics to perform specific tasks for human health. For example, an engineered *Lactococcus lactis* has been used to treat type II diabetes [5], [6]. Additionally, an engineered *Escherichia coli* Nissle 1917 (*E. coli*) is being tested in a phase II clinical trial (NCT04534842) based on its ability to breakdown phenylalanine to treat phenylketonuria after demonstrating efficacy in animal studies [7]. Further attempts are made to combat pathogenic bacteria, such as *Pseudomonas aeruginosa, Vibrio cholerae and Salmonella* using engineered *E. coli* Nissle strains [7].

Numerous studies have also demonstrated a link between skin health and gastrointestinal health [8]. Gastrointestinal disorders are often associated with skin disorders: 7–11% of patients with inflammatory bowel disease also suffer from psoriasis [9]. For example a high-carbohydrate diet has been linked with acne vulgaris [10] and dandruff [11]. Disbalances in the gut microbiome appear to contribute to common Western skin conditions [12]. As an altered gut microbiome has been found in many common skin conditions, such as acne [13] atopic dermatitis [14], [15] and rosacea [16]. The Western diet, which is rich in fat and carbohydrates and low in fibre, has altered the gut microbiome, which can lead to an impaired mucus layer and, in further stages, an impaired intestinal barrier. This contributes to an inflammatory state in the body that can be expressed as a skin pathology [17]. Probiotics and diet manipulations have been successfully applied to (partly) restore skin disorders [18]. The gut-skin axis is not only governed by diet, and even acts bidirectionally with UVB and topical allergies directly influencing the gut [19], [20], [21]. Therefore, the cross-talk between the gut and skin is an important factor to take into consideration in regard to skin disorders.

While the research is the most advanced in the gut microbiome, the microbiomes of other body parts, such as the skin, vaginal, oral, lung and even the eye, have caught the attention of the research community. Given that the skin is the largest organ of the human body, it functions to separate the environment from the internal compartments of the human body and is directly involved in water retention and protection from pathogens and toxins [22]. Similar to the gut, the skin forms a barrier between the internal organs and the outside. The skin is a unique microenvironment where its inhabitants need to counterbalance many challenges, including: sunlight, temperature shifts, and moisture. In addition, the skin has its own immune system, represented by keratinocytes and sebocytes which can take part in innate immune functions [23]. This part of the skin immune system is in a tight interplay with its microbiota. It is constantly reacting to external factors with immune response signals but also influencing the composition of the skin microbiota through the secretion of antimicrobial peptides (AMPs) by human cells like sebocytes or bacterial cells [24], [25], [26], [27], [28]. For example the host can control which species colonise certain ecological niches such as the follicle [29]. At the same time bacterial species of the skin can synergistically act with the host to eradicate other species [27]. As well as the metabolites of the bacteria can influence the host to resolve inflammation [30] or modulate its basic immune response [31].

Although we tend to speak about the skin microbiome, the populations of microorganisms differ vastly between different skin areas. It is technically challenging to manipulate or sample locally in the gut, but the skin represents an easily accessible organ for such studies. It is therefore a prime environment for manipulation of the microbiome.

## 2 The skin microbiome

The human skin is divided into moist, dry and sebaceous sites. Each of these sites has its own microorganism ecosystem but are similar at the species level. All these sites share *Cutibacterium, Corynebacterium and Staphylococcus* species on the bacterial level and *Malassezia* species on the fungal level in different distributions [32]. The sebaceous sites are predominated by cutibacteria and staphylococci*,* while the moist sites are predominated by *Corynebacterium* and *Staphylococcus* species [33]. The dry skin parts contain the lowest amounts of bacteria, but the diversity is the highest, including *Corynebacterium*, *Cutibacterium*, *Staphylococcus*, and a wide range of Proteobacteria [32].

The skin microbiome is relatively stable over time. The same microbiome comes back after washing and cosmetic usage, even when those cosmetics contain antimicrobial agents [34], [35]. Zeeuwen et al. [36] showed by means of tapestripping that the skin microbiome is actually derived from within the skin. Up to 14 days after skin removal, the newly developed skin microbiome was more similar to that of the deeper stratum corneum layers compared with the initial surface microbiome. Additionally, Nakatsuji et al found higher amounts of bacterial DNA in hair follicles than on skin epidermis [37], [38]. This and another study found that bacterial DNA was even present in the dermis and adipose tissue, although no evidence was given whether these bacteria were also alive [37], [38]. Altogether, these findings lead to the hypothesis that the microbiome of the deeper layers is the core skin microbiome.

Interestingly, the dynamics of the skin microbiome vary depending on the skin site. Sebaceous and moist skin sites exhibit a very stable composition over time. Dry skin sites are more unstable partly due to the low number of bacteria present, limited nutrients available, and more frequent external influences [39].

The human skin has been divided based on its topography, since different skin sites are favoured by certain microorganisms [33], [40]. While the oily forehead harbours a certain population, the dry elbow harbours another. Biogeography and individuality of the healthy skin microbiome was shown to vary both in its microbial abundance or diversity but also in their functionality. Metabolic diversity was correlated with the diversity on species level. For example, sebaceous sites have low species diversity and have been shown to have lower metabolic function compared to other body sites. When comparing heterogeneity of one species on different skin sites it could be seen that, for example *Cutibacterium acnes* (*C. acnes*) were specific on individual level but exhibited less site-specificity. *Staphylococcus epidermidis* (*S. epidermidis)* in contrast was site-specific and similar between individuals [40].

The biogeographical differences play a role in microbial, fungal or viral stability on human skin and have provided insights into multiple site-specific skin conditions.

The advancement of sequencing technologies stressed the importance of strain level differences of the microorganisms. It has been shown that some strains of the same species can be beneficial and others pathogenic to the host. Usually, in disease, not only one strain drives the pathogenicity but the change of abundance of specific strains or organisms [41], [42], [43], [44].

## 3 Dysbiosis of skin: Diseases related to dysbiosis

As the microbiome differs topographically, dysbiosis of skin occurs topographically on specific skin sites in a manner similar to that noted in atopic dermatitis, rosacea, acne vulgaris and psoriasis [45], [46], [47].

Interestingly, psoriasis and atopic dermatitis are both diseases with a strong influence of the immune system and commonly occur at dry skin sites. Despite being distinct diseases, the corresponding dysbiosis of the microbiome at a global level is very similar between the two diseases (increase in *Staphylococcus aureus (S. aureus),* decrease in *C. acnes* and other commensals of the skin) [48].

Atopic dermatitis is an autoimmune disease driven by an overexpression of IL-24 and IL-13 cytokines, and the link between this condition and the microbiome has been described. Atopic dermatitis has been more intensely studied, and we understand now that the strain level differences of the *Staphylococcus* population seem to be strongly associated with atopic dermatitis flares. *S. aureus* abundances are increased during disease flares [49]. When atopic dermatitis skin is treated with biologicals that suppress the immune response, *S. aureus* abundance (both relative and absolute counts) is reduced, resulting in clinical improvement [50].

Psoriasis is a typical autoimmune disease that is driven by an overexpression of IL-23 and IL-17 cytokines, but no direct link has been found between skin microbiome imbalance and disease pathology [51]. However, the involvement of fungi was recently suggested [52]. A more thorough investigation of the microbiome is needed.

Acne vulgaris is another widespread skin disease that typically affects sebaceous skin areas in contrast to the previously mentioned conditions. In this case, the dysbiosis of the microbiome has been associated with certain strains of *C. acnes*. Multiple genetic markers to distinguish between health and disease associated strains have been suggested over recent years and are reviewed elsewhere [46].

Dandruff is a pathology on the scalp that is generally associated with a fungal component. Particular fungi (*Malassezia furfur* and *Malassezia globosa*) present on the scalp are thought to cause an overproduction of oleic acid, which disturbs the stratum corneum cells and evokes an inflammatory response on the scalp [53]. Recently, a bacterial impact was also suggested based on an imbalance in *Cutibacterium* and *Staphylococcus* species [54].

Although not usually recognized as pathology, (heavy) body odour is known as a bothersome condition [55]. The link between this condition and the microbiome has been clearly described. A higher proportion of *Corynebacterium*, *Anaerococcus*, *Peptoniphilus*, and *Staphylococcus hominis* (*S. hominis*) causes more malodorous volatiles from apocrine sweat, particularly in people with the CG or GG allele in the ABCC11 gene [56].

Rosacea has also been linked to a dysbiosis of the skin microbiome. An increased abundance of *Demodex* mites are observed in this disease. An interesting suggestion was made by Parodi et al. who reported an interplay between the skin and bacterial overgrowth in the small intestine [47], [57]. Rosacea patients had a significantly higher overgrowth of gut bacteria than controls and elimination of the overgrowth, using an antibiotic, resulted in an almost complete regression of the skin pathology for a prolonged time. These findings support the pathogenetic role of the gut microbiome in rosacea lesions, although the exact relationship remains to be elucidated [58]. Additionally, research even investigated the microbiota of the *Demodex* mites, but final conclusions are still outstanding [59].

Even skin cancers, such as squamous cell carcinoma, and its predecessor condition actinic keratosis are associated with dysbiosis of the skin microbiome [60]. As noted with other lesional skin diseases, an increase in *S. aureus* is observed in combination with a decrease in skin commensals, such as *C. acnes*. Recent research discovered a potential protective mechanism of *C. acnes* against UV-induced reactive oxygen species (ROS) [61]. In a follow-up study, the authors showed that the protective enzyme is indeed reduced in actinic keratosis and basal cell carcinomas [62]. Other work discovered that specific *S. epidermidis* strains can selectively inhibit the proliferation of tumour cell lines [63]. This finding enables the exciting hypothesis that skin commensals, such *C. acnes* and *S. epidermidis*, protect the host from UV-induced DNA damage in a symbiotic relationship.

However, in all of the above-described cases, despite impressive evidence, we cannot completely distinguish whether the observed microbiome dysbiosis is a cause or consequence of the disease. The only way to concisely answer this question is by directly changing a diseased microbiome to the proposed healthy state. If, as a consequence of this change, the disease improves, only then we can truly assume a causative relationship between microbiome dysbiosis and the disease.

The skin is an ideal area for such experiments. Theoretically, the existing microbiome can be reduced with topical disinfectants on locally defined areas. New bacteria can then be applied, and their behaviour can be monitored. Multiple studies aiming to change the skin microbiome have already been performed and delivered encouraging results. A main concern for each such study is the safety of the subjects, which needs to be evaluated on a case-by-case basis.

## 4 Skin microbiome manipulation strategies

The skin microbiome can be changed via a multitude of mechanisms. The first method is a skin microbiome transplant. Microbiome transplantation for humans is not new and is best known by the example of faecal microbiota transplantation (FMT) for the treatment of gastric *Clostridium difficile* infections [64], [65]. In a skin microbiome transplant, the skin microbiome of a healthy individual is transferred to the washed and/or disinfected skin area of another person with the aim of improving the skin condition of the latter. The advantage is that the microbiome is transferred in its natural environment. Although straightforward, this method has several disadvantages. Only a low number of bacteria can be harvested from a person’s skin. A culturing step is typically necessary to obtain sufficient amounts of bacteria. The method is not scalable or industry applicable. It is not immediately clear which bacteria, fungi or viruses are transmitted to the person's skin. As such, potential pathogenic taxa can also be transmitted.

The second method is by means of skin bacteriotherapy, where one or multiple pure cultures with health-promoting properties are placed on the washed and/or disinfected skin area of a person. The applied microbiota can be (1) alive (probiotics): a probiotic is a living microorganism that, when added in sufficient amounts, exerts a beneficial effect on the host [66]. (2) Tyndallized or thermokilled bacteria (postbiotics): bacterial cell structures, enzymes and excreted bacterial factors are added, but the bacteria do not replicate anymore. (3) Cell lysates or physically killed bacteria (postbiotics): the bacteria are destroyed, and the cell contents and cell walls are in solution. The bacteria do not replicate anymore, but the enzymes can still be active. (4) Purified enzymes: single or groups of bacterial enzymes are purified and added. (5) Fermentation products or supernatants: the bacteria are not added, but the supernatants containing their antioxidants, amino acids, lipids and/or vitamins are added. The methods 1–5 have multiple advantages over a skin microbiome transplant with the main advantage being that the process is easier scalable and thus industrial applicable. For method 1 (application of live probiotics), highly concentrated bacteria can be applied; thus, a higher efficacy can be obtained compared to a complete skin microbiome transplant. Pro- or postbiotics can be applied in a skin emollient, creme or suitable medium for skin. There are also a series of drawbacks associated with the use of pro- and postbiotics. Bacteria are cultured in sugar-rich media; it can therefore be more difficult for the bacteria to adjust to a sebum-rich environment. Skin engraftment is not easy; the applied bacteria compete with the skin resident microbiome of the deeper skin layers. The application of high amounts of bacteria could lead to a skin immune reaction with irritation and side effects as a result.

A third method of changing the skin microbiome is through prebiotic stimulation. In this process, prebiotics are supplemented to the skin to stimulate the growth of specific health-promoting microbes. A prebiotic is an ingredient with a bioselective activity that exerts a beneficial effect on the host and attempts to improve the host's health [67]. There are several advantages to this method. There is no need to work with living bacteria; thus, there is a reduced chance of a skin immune reaction. The method has an indirect mechanism of action. Prebiotics are typically well-defined compounds for which side effects are well studied. The INCI name and safety sheets are normally available. There are also disadvantages. The indirect method has less direct results. Prebiotics could also stimulate non-targeted low-abundance bacteria. The effect of prebiotics can be unpredictable given the variability in the skin microbiome, physiology and immune response in different individuals.

All methods have their advantages and disadvantages. Scientific research is currently being conducted using several of these methods to treat common skin disorders.

## 5 Human skin microbiome manipulation efforts

### 5.1 Transplanting microbes from one body site to another within the same subject (Costello et al. 2009 [68])

Costello et al. raised the question of whether changes in microbial communities are due to environmental factors or due to historical exposures. To answer this question, bacterial communities were transplanted from the tongue to the forehead or volar forearm or from the volar forearm to the forehead or tongue. Samples were taken after 2, 4 and 8 h. Tongue to forearm transplantation led to an engraftment of tongue bacteria, whereas tongue to forehead transplantation did not lead to a great change in the original native microbiome composition. Similar to this finding, forearm to forehead transplant or vice versa exhibited communities similar to the native state (Table 1) [68].

**Table 1:** List of skin microbiome modulation studies to date.

Skin microbiome modulation	Study outcome	Applied Microbiome composition	Reference or clinical trial identifier
Microbiome from non-smelly siblings was applied on odourous siblings	Reduction of axillary malodour	Enriched in *Staphylococcus* and less rich in *Corynebacterium*	Callewaert et al. 2017 [77] NCT01581112, NCT01944566
Application of *S. epidermidis* strains	Reduction of axillary malodour	*S. epidermidis*	NCT03967470
Application of own *S. epidermidis* strains	Relative increase in water and lipid content, decrease in water evaporation and pH value	*S. epidermidis*	Nodake et al. 2015 [76]
Application of AMP producing coagulase-negative *Staphylococcus* (CoNS)	Decrease in *S. aureus* abundance	*S. hominis* or *S. epidermidis* isolated from AD patient’s skin	Nakatsuji et al. 2017 [27] NCT03158012, NCT02144142, NCT03151148
Application of combination of *C. acnes* SLST types H1 + A1 + D1 on healthy individuals	Applied *C. acnes* mixture engrafts on human skin	*C. acnes* strains isolated from healthy individuals	Paetzold et al. 2019 [83]
Application of combination of *C. acnes* strains on acne vulgaris patients	Applied *C. acnes* mixture shifted towards formulation, non-inflamed lesions, comedone count and pH reduced	*C. acnes* strains from healthy individuals	Karoglan et al. 2019 [82]
Microbial transplant from tongue to forehead or forearm, from forehead to forearm or vice versa	Tongue bacterial community engrafted on forearm but not on forehead. Forehead or forearm transplants showed similarity to initial state	Whole microbial communities from tongue, forehead or forearm	Costello et al. 2009 [68]
Axillary bacteria transferred on forearm	Lipophilic and large colony diphtheroids with apocrine sweat produced strong body odour on subjects' forearms	Single axillary bacteria, excluding *Propionibacterium*	Leyden et al. 1981 [74]
Transfer of microbiome between dissimilar non-autologous environments (arm to upper back)	After 24 h, a median of 4 arm-only species were still present at the transplanted area	Complete naive superficial microbiome	Perin et al. 2019 [74], [75]
Addition of *Nitrosomonas eutropha* to subjects with various skin disease	11 clinical studies on skin and other diseases. Data only publicly available for one phase II trial in acne vulgaris with a positive outcome	*Nitrosomonas eutropha* B244	Topical application: NCT02656485 NCT02832063 NCT03235024 NCT03775434 NCT03268174 NCT03590366 NCT04490109 NCT03243617
Ascending dose of heterologous *S. epidermidis*	*S. epidermidis* for skin appearance	Proprietary *S. epidermidis* strain AZT-04	NCT03820076
Application of *C. acnes* strains with DeoR repressor to acne vulgaris patients	Safety endpoint reached and improvement in inflamed lesion count compared to placebo	NB-01 proprietary *C. acnes* strain	NCT03709654 NCT03450369
Application of *Lactobacillus* strains to treat acne vulgaris.	Two studies conducted but no data publicly available	Mix of established gut probiotics (*Lactobacillus pentosus, L. plantarum* and *L. rhamnosus*)	NCT03469076 NCT04216160

### 5.2 Transplanting skin microbes from one skin site to another within the same subject

Multiple publications have shown that syntrophy is important to maintain the metabolic interplay between species [69], [70], [71], [72], [73]. While most studies focus on the transfer of just one phylogenetic group of bacteria without taking into account a potential cross-feeding, a couple of studies were performed where the whole naive skin microbiome was transferred from one skin site to another.

Leyden et al. (1981) tested the transfer of underarm odour-causing bacteria to the forearm of subjects to verify the reproduction of the malodour. The samples with the two types of diphtheroids incubated on the forearm produced a strong odour, demonstrating that the odour-causing bacteria could be transferred from the armpit to the forearm (Table 1) [74].

Perin et al. (2019) transferred microbiome swabs from the arm to the upper back of the same person. The microbiome composition of the antecubital fossa (inner elbow) exhibited increased diversity in contrast to the back. While the back was mainly inhabited by the abundant *Cutibacterium*, the inner elbow showed comparable amounts of *Staphylococcus, Streptococcus,* and *Corynebacterium*. Despite the fact that sampling and transfer of the complete community was difficult, especially Gram-negative species, a median of 4 arm-only species were still present in the transplanted area after 24 h. These genera were mainly from the taxa *Gardnerella, Brachybacterium, Janthinobacterium, Actinomyces, Anaerococcus, Microbacteriaceae,* and *Dermabacteriaceae* (Table 1) [75].

### 5.3 *S. epidermidis* application on facial skin (Nodake et al, 2015 [76])

In a double blinded randomized clinical study, *S. epidermidis* strains were first isolated from individuals. The isolated strains were then cultured and applied back to the same subjects' facial skin twice per week for a duration of 4 weeks in total. Compared to the control group, *S. epidermidis* application increased the relative lipid and water content but decreased water evaporation from the patient's skin. Additionally, skin acidity measured as pH was reduced to 5 from 5.5 in patients with applied *S. epidermidis*. This decrease in acidity could be due to the increase in lactic acid and propionic acid in the patients. This study showed the beneficial impact of *S. epidermidis* application on the face of human skin and its potential as a cosmetic ingredient (Table 1) [76].

### 5.4 Armpit microbial transplants to treat body odour (Callewaert et al, 2017 [77])

In a first of its kind clinical study, a series of siblings of which one exhibited strong body odour were enrolled in this study [77]. Bacteria play an important role in body odour; thus, the skin microbiome of the non-smelly sibling was successfully established on the sibling with strong body odour. A trained odour panel could detect a reduction in body odour coupled to a new equilibrium of microbiota that was richer in staphylococci with less corynebacteria. To improve the establishment of the applied strains, the skin of the recipient was disinfected before application of the new bacteria. The application of pure cultures of *S. epidermidis* also resulted in better odour scores than before (unpublished data) (Table 1).

### 5.5 Bacteriotherapy to treat atopic dermatitis (Nakatsuji et al, 2017 [27])

*S. aureus* has been linked to dysbiosis in AD patients, and it has been proposed that deficiency in antimicrobial peptides (AMPs) produced by skin cells could be linked to the loss of protection against *S. aureus* growth [78], [79]. Nakatsuji et al. showed that members of the healthy microbiome can provide selected protection against *S. aureus* by secreting Sh-lantibiotics. Sh-lantibiotics are AMPs which are similar to other detected lantibiotics which show bactericidal modes of action to inhibit different species [27], [80]. In this study, the microbiome of lesional skin was compared to the microbiome of patients colonized or non-colonized by *S. aureus* and found that those colonized by *S. aureus* were less diverse in taxa. Additionally, they found 10-fold more relative CFU based on DNA abundance in contrast to life colony counting in normal skin, and they concluded that this finding could be related to an antimicrobial defence system that was not active in lesional AD patients. Since coagulase-negative *Staphylococcus* (CoNS) (*S. epidermidis* and *S. hominis*) exhibits the potential to produce AMPs, they isolated CoNS strains from subjects with atopic dermatitis that were able to inhibit *S. aureus* growth. These strains were then amplified and subsequently applied to the skin of the subjects (autologous transplant). A reduction in *S. aureus* colonization was reported, but the clinical symptoms were not measured (Table 1) [81].These findings lead to the creation of the company Matrisys Bioscience and is currently being developed further. The aim is to obtain a single strain that can be applied to many patients.

### 5.6 Skin microbiome modulation to treat acne vulgaris (Karoglan et al., 2019 [82])

Paetzold et al. isolated whole microbiome samples of two individuals but also used mixtures of specific *C. acnes* species to test their transplantation. In addition, the synergistic effects of species were also considered. These samples were transplanted on different individuals on sebaceous-rich skin sites. Analysis showed five different dermatotypes of *C. acnes*. Engraftment occurred after only three days and could be observed even after application has been stopped for many days. Transplantation of specific mixtures was engrafted better than whole microbiome samples with a concentration of 10^8 CFU/mL. Additionally, mixtures of multiple species engrafted best over single *C. acnes* species. Donor skin microbiota containing more *C. acnes* species than *Staphylococcus* species appeared to be more suitable for transplantation (Table 1) [83]. In an extension of this experiment, Karoglan et al. (2019) applied mixtures of *C. acnes* strains to subjects with acne vulgaris. The autochthonous skin microbiome was first reduced using benzoyl peroxide treatment. After this initial disinfection, a bacterial mix of two and four live *C. acnes* strains was applied twice per day for 5 weeks. In this open label study, a statistically significant reduction in lesion count was obtained. The clinical relevance of this finding remains to be proven in a double blinded randomized placebo controlled trial. However, the applied bacteria could be detected after the treatment in ~50% of the study participants (Table 1). Surprisingly, no advantage in terms of the engraftment effect of the four strains over the two strain solutions was detected. These findings are currently further developed by the company S-Biomedic.

### 5.7 Application of non-commensal bacteria on skin

AOBiome has conducted a series of clinical trials with their lead strain *Nitrosomonas eutropha* (Table 1). This bacterial species is normally not found on normal Western skin. However, it is argued that we lost this species, which is normally predominantly found in the soil and sewage plants due to increased hygiene. Positive results of a phase IIb trial were announced in a press release but have not yet been publicly posted at ClinicalTrials.gov. The results of additional trials in pruritus associated with atopic dermatitis and rosacea were started, and results should also become available soon.

### 5.8 Genetically modified bacteria as topical therapeutics

An interesting approach is the genetic engineering of skin commensals such, *S. epidermidis* or *C. acnes*, to produce and secrete active biotherapeutics. Possible biotherapeutics include Fillaggrin, LEKTI, IL-10, anti-inflammatory somatotropin or other growth factors and hormones. The company Azitra already tested their bacterial chassis organism in a phase I clinical trial, and the results are expected soon. The company ILYA is also running a phase I clinical trial using *Lactobacillus reuteri* as a chassis that secretes CXCL12, a short-lived human cytokine, to improve wound healing. The main advantage of this approach is that an active agent with a short half-life can be efficiently delivered to the site of action.

### 5.9 Application of *Lactobacillus* and other gut probiotics on skin

In recent years, many companies have started to incorporate established probiotics or derivatives thereof in products for topical application. In 2009, a first group investigated the use of *Lactobacillus plantarum* in wounds [84]. Whether this acts through similar or different pathways than recent results [85], [86] remains to be elucidated. At the same time, many cosmetic companies started using probiotic derivatives, such as extracts or postbiotics. Lactobacilli are an interesting group of bacteria given their proven safety and long use as probiotics for the gut. However, to date, only a few companies took the technical challenge to also incorporate live bacteria in their product.

## 6 Discussion and outlook

Changing the skin microbiome by applying live bacteria has gained significant interest. There are increasingly more associations between specific microbial species and skin diseases. Many studies do not completely address the cause or consequent conundrum. To obtain a valid answer to this question is challenging; it can only be achieved by changing a diseased state microbiome to the proposed healthy state. Given its easy access and great safety profile, the skin microbiome is one of the best sites to answer such questions. Bacteria can be easily applied to locally defined areas. In the case of adverse events, the applied bacteria can be removed with a disinfection or systemic antibiotic treatment.

One of the major challenges in manipulating the skin microbiome is to make the applied bacteria stable on skin. Despite initial topical disinfection steps, it is very difficult to remove the subcutaneous microbiota. Therefore, the applied new microbiota on the skin epidermis will be in competition with those of the deeper skin layers.

This phenomenon, which called engraftment, is not easily achieved and is also a major hurdle for researchers in the gut microbiome a major hurdle. So far only few studies are publicly available which reported on the interpersonal strain transfer. Their data indicate that different strains could potentially work synergistically together [83] or exclude each other [28]. One very interesting variant of strains working synergistically together is that the continued application of one or more live strains can cause a new stable community to emerge in which the probiotic OTU(s) being relatively abundant while synergising with the pre-existing strains. In each case, the numbers of subjects accepting the new strains were low, and a clear reason was not identified. More intense research in this direction is needed to better predict potentially engrafting subjects. An interesting approach could be to couple the classic culture-independent analysis with a culture-based approach. The isolated cultures could then be screened against each other to detect their ecological interplay.

Once the interactions of the bacteria with each other are fully understood, we can address the microbiome host interplay. In one of our earlier studies, we identified widespread strains that are easier to establish on the skin. Whether this finding indicates that they are metabolically fitter than other strains or are more tolerated by the host is unclear. More systematic studies would be desirable to better understand whether indeed every host is only tolerating a certain subset of strains or whether any given bacterial strain that is native to the given environment can be planted on the skin.

When using pure cultures, another challenge lies in the culturing of the microbiota. Microbiota are typically grown in sugar-rich media. Then, the bacteria are lyophilized and covered in a sugar coating. These microbiota are adapted to the sugar-rich environment where they were cultured. When the bacteria are applied on someone’s skin, which is very poor in sugars and nutrients overall and richer in lipids, the bacteria have difficulties adapting to the new environment. In designing such experiments, it is necessary to choose the appropriate culture media so that the applied microbiota can easily colonize the skin environment.

In the past 100 years, with the industrial revolution, we have drastically adapted our lifestyle with a coupled increase in autoimmune skin conditions. Skin conditions are commonly treated with antimicrobial or anti-inflammatory approaches. Although these approaches may be successful in the short run, antimicrobial approaches ultimately lead to disbalances and an increase in multiresistant strains. Anti-inflammatory approaches can locally alleviate the problem, but the solution is often found by in depth systems biology studies.

The Western skin microbiome has lost considerable diversity compared to indigenous skin [87]. The Western hygiene habits, including frequent use of skin cosmetics and detergents and associated removal of skin lipids, have led to a change in the skin microbiome. Indigenous, non-urbanized people and farmers carry a considerably more diverse skin microbiome and are less prone to skin allergies, acne and other skin disorders [88]. Such lifestyle and environment can thus be important to maintain a healthy skin microbiome. However, many Western people have no skin conditions. Therefore, there is value in studying their microbiome to understand whether it is better adapted to a Western lifestyle than others. There is likely no easy answer to be found, but such research could answer many unresolved questions.

Most of the research performed has focused on bacteria. Bacteria are nonetheless not the only inhabitants of human skin. Additionally, fungi, phages and micro-eukaryotes are present on the skin and most likely play a role in normal skin homeostasis. Known examples are *Malassezia* in dandruff / seborrheic dermatitis, *Demodex* in rosacea and even a possible fungal influence in psoriasis. The manipulation of these inhabitants can also be valuable in addressing skin conditions and more research in this field is needed.

Despite the challenges and hurdles to overcome, a bright future is laid ahead for skin microbiome modulation in the treatment of skin conditions. The first available study outcomes are already very promising. In the coming years, we are expecting much scientific data to become available from the clinical trials currently being performed. The skin microbiome is very important for skin health; therefore, the presence of the “good” species is of utmost importance in protecting the skin and maintaining the skin in a healthy state.

## CRediT authorship contribution statement

**Chris Callewaert:** Writing - review & editing. **Nastassia Knödlseder:** Writing - original draft. **Ante Karoglan:** Writing - review & editing. **Marc Güell:** Writing - review & editing. **Bernhard Paetzold:** Writing - review & editing, Funding acquisition.

## Declaration of Competing Interest

All authors of this review previously published in this field. MG and BP are founders of S-Biomedic N.V. a company active in the skin microbiome field.
